# Social determinants of stage IV anal cancer and the impact of pelvic radiotherapy in the metastatic setting

**DOI:** 10.1002/cam4.1203

**Published:** 2017-10-04

**Authors:** Michael C. Repka, Nima Aghdam, Andrew W. Karlin, Keith R. Unger

**Affiliations:** ^1^ Department of Radiation Medicine Georgetown University Hospital Washington Washington DC

**Keywords:** Anal cancer, metastatic disease, NCDB, radiotherapy, social determinants of health

## Abstract

Anal cancer is a relatively rare malignancy, and a minority of patients present with metastatic disease in the United States. The National Cancer Database (NCDB) was used to identify factors associated with metastatic disease at presentation and evaluate the role of pelvic radiotherapy in these patients. The NCDB was queried for patients with squamous cell cancer of the anus diagnosed between 2004 and 2013. Patients were stratified by clinical stage at diagnosis, and a binary logistic regression model was created to identify factors associated with metastatic disease at diagnosis. A secondary metastatic cohort was generated and a multivariable Cox proportional hazards model was created to identify factors associated with improved survival. To validate findings, propensity‐score matching was performed to generate a 1:1 paired dataset stratified by receipt of pelvic radiotherapy. The primary analysis cohort consisted of 28,500 patients. Facility location, male gender, and lack of insurance were confirmed as independent risk factors for metastatic disease. The metastatic cohort consisted of 1264 patients. Multivariable analysis confirmed female sex, possession of a private or Medicare insurance plan, pelvic radiotherapy, and chemotherapy as independent predictors of improved survival. A propensity‐score matched cohort of 730 patients was generated. The median survival was 17.6 months in patients who received radiotherapy versus 14.5 months in those who did not (*P *< 0.01). In this cohort, male gender and lack of insurance were associated with metastatic disease at presentation. Furthermore, a significant benefit was associated with the use of pelvic radiotherapy. Future prospective research is warranted to confirm these findings.

## Introduction

Accounting for just 0.5% of all cancer diagnoses with an estimated annual incidence of 8,200 in the United States, anal cancer is a relatively rare malignancy 1. The majority of patients present with clinically localized disease amenable to curative treatment, which typically consists of organ preserving chemoradiotherapy with radical resection reserved for local progression or treatment failure 2. However, a small proportion of patients will present with metastatic disease at the time of initial presentation. Although no routine screening is recommended for the detection of anal cancer, early bothersome symptoms, which can include bleeding, loss of continence, or discovery of mass, are potentially responsible for the low rates of metastases at diagnosis 3, 4.

The evidence that socioeconomic factors play a role in determining health outcomes has gained considerable traction over the past several decades. Particularly, clear links have been demonstrated between socioeconomic factors (e.g. ethnicity, income, and insurance status) and health outcomes for patients with diabetes 5. Despite years of research and attempted interventions, these discrepancies have persisted well into the 21st century. While not as thoroughly investigated, the available evidence suggests that similar social factors may affect patients with a variety of malignancies 6, 7. A more thorough understanding of social determinants of health outcomes in cancer patients is critical. However, with the limited number of cases of anal cancer diagnosed each year, conclusions can be difficult to draw utilizing institutional databases and similar sources of information.

The National Cancer Database (NCDB) is a prospectively collected, hospital‐based database which captures approximately 70% of all cancer diagnoses in the United States 8. Initiated in 1988, the NCDB is a joint project of the Commission on Cancer of the American College of Surgeons and the American Cancer Society. All patient information is collected and entered into the database without personal identifiers, and specific disease site databases are distributed to NCDB researchers in the form of a completely deidentified participant user file (PUF). While studying the NCDB can be useful for many malignancies, it is particularly suited to situations where conducting prospective trials is not feasible. Furthermore, this database can be analyzed to ascertain information not typically gleaned from prospective trials, such as epidemiological data or discrepancies in patterns of care.

While the optimal treatment paradigm for localized anal cancer has been investigated in several large prospective clinical trials 9, 10, 11, 12, there are scant data regarding the role of local therapy in the treatment for metastatic patients. Indeed, statements from major national organizations, such as the National Comprehensive Cancer Network (NCCN) guidelines, are extrapolated from the recommendations for patients with localized disease. However, evidence exists which suggests multidisciplinary treatment may prolong survival in select patients presenting with metastatic anal cancer 13, and a recent clinical trial demonstrated a survival benefit for patients with metastatic squamous cell carcinoma of the esophagus treated with local radiotherapy 14. In this study, the NCDB was analyzed to identify patient‐related factors associated with metastatic disease at presentation and evaluate whether the addition of pelvic radiotherapy provides a survival benefit to patients with metastatic anal cancer.

## Material and Methods

This study was granted an exemption from the Georgetown University Institutional Review Board (IRB, study number 2017‐0577). The NCDB was queried for patients aged ≥18 years old with cancer of the anus (ICD‐10 codes C21.0, C21.1, and C21.8) diagnosed between 2004 and 2013. The 7th edition American Joint Committee on Cancer (AJCC) Staging Manual was used to assess the patient's clinical stage. Patients were excluded from the analysis in the setting of nonsquamous histology, noninvasive disease (e.g. AJCC clinical stage 0), or if the AJCC clinical stage was unknown or not recorded. Patients were then excluded if the presence of social determinants (insurance status, education, income, population density) and hospital factors (facility region, facility type) were not recorded or unknown to create a primary cohort for analysis. Education and income are reported indirectly to the NCDB; each measure is estimated by the average educational attainment or annual income of the patient's zip code. These values are reported in the NCDB as equally proportioned quartiles derived from 2012 American Community Survey data. Educational attainment is estimated by the number of adults in each zip code who do not obtain a high school diploma. The Charlson–Deyo Comorbidity Index was used to assess patient comorbidities 15, 16; patients were classified as have a score of 0, 1, or ≥2.

Patients were then stratified by clinical presentation as localized (AJCC clinical stages I–III) or metastatic (AJCC clinical stage IV), and the Pearson chi‐square test was used to identify statistically significant associations between categorical variables and metastatic disease at the time of diagnosis. Statistically significant factors (*P* < 0.05) from this analysis were included in a multivariable binary logistic regression model to determine independent risk factors associated with metastatic disease at the time of diagnosis. All findings with a *P* < 0.05 were considered statistically significant in the multivariable model.

Following the initial analysis, a secondary metastatic cohort was generated by excluding patients with clinical AJCC stages I–III, unknown follow‐up, or unknown receipt of chemotherapy, radiotherapy, or surgery. Patients were stratified by receipt of pelvic radiotherapy, and chi‐square analysis was used in order to assess the significance of differences between patient groups. Overall survival (OS) analysis was performed using the Kaplan–Meier method. Univariate analysis of patient and disease‐related factors were performed using the Cox proportional hazards method, and statistically significant findings were included in a multivariable Cox proportional hazards model. To validate findings from this analysis, propensity‐score matching was performed using the nearest neighbor algorithm with a caliper width of 0.2 to generate a 1:1 paired dataset for further analysis. A Chi‐square analysis was used to confirm even distribution of patient groups, and the Kaplan–Meier method was used to analyze overall survival in the paired cohort. All findings with a *P* < 0.05 were considered statistically significant. SPSS Statistics Version 23.0 (IBM Corp., Armonk, NY) was utilized for data management and analysis.

## Results

**Figure 1:**
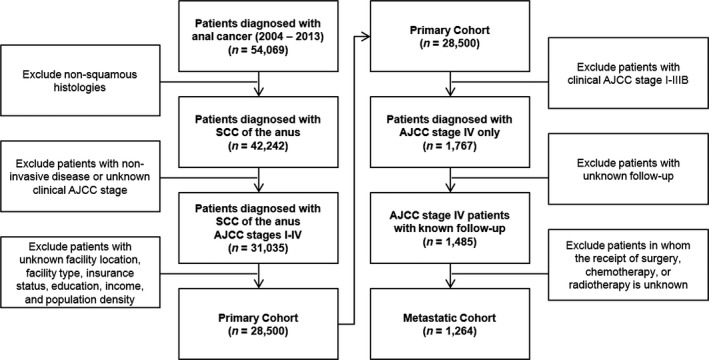
CONSORT schema.

**Figure 2:**
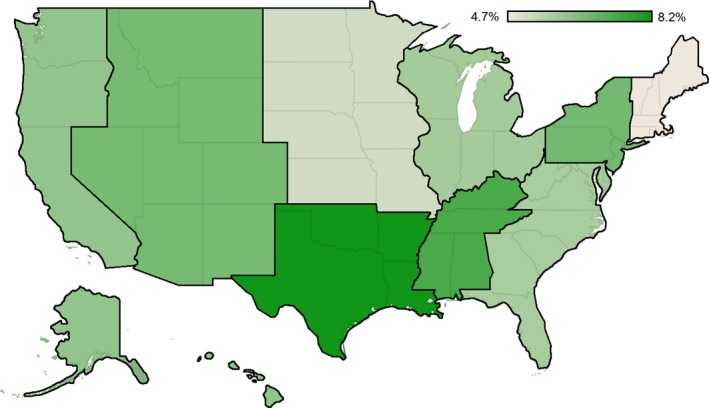
Regional variation in incidence of metastatic disease at diagnosis in patients with squamous cell carcinoma of the anus.

The initial cohort including all patients comprised 54,069 patients; following application of exclusion criteria the primary cohort consisted of 28,500 patients (Fig. 1). Factors associated with a diagnosis of metastatic disease at presentation by simple proportional analysis included facility type, facility location, gender, median income, education, and insurance status (Table 1). These factors were then incorporated into multivariable logistic regression model which confirmed facility location and male gender as independent risk factors for metastatic disease at presentation (Table 2). Compared to patients without insurance, those who held private or Medicare insurance plans were less likely to present with metastatic disease. Intriguingly, there was a nearly twofold higher incidence of metastatic disease at presentation in patients treated at facilities in the West South Central region (Texas, Arkansas, Oklahoma, and Louisiana) compared to the region with the lowest incidence, New England (Maine, Vermont, New Hampshire, Massachusetts, Connecticut; Fig. 2). Facility type, median income, and education were not associated with a stage IV diagnosis in the adjusted model.

**Table 1:** Baseline patient characteristics for the primary cohort, stratified by AJCC stage at diagnosis

	Stages I–IIIB (%)	Stage IV (%)	*P‐*value
Facility type			0.016
Community Cancer Program	2997 (11.2)	204 (11.5)	
Comprehensive Community Cancer Program	12067 (45.1)	734 (41.4)	
Academic/Research Program	8845 (33.1)	643 (36.4)	
Integrated Network Cancer Program	2824 (10.6)	186 (10.5)	
Facility location			<0.01
New England	1558 (5.8)	77 (4.4)	
Middle Atlantic	3920 (14.7)	281 (15.9)	
South Atlantic	6051 (22.6)	375 (21.2)	
East North Central	4734 (17.7)	297 (16.8)	
East South Central	1794 (6.7)	142 (8.0)	
West North Central	2005 (7.5)	110 (6.2)	
West South Central	1657 (6.2)	148 (8.4)	
Mountain	1256 (4.7)	89 (5.0)	
Pacific	3758 (14.1)	248 (14.0)	
Gender			<0.01
Male	8643 (32.3)	633 (35.8)	
Female	18090 (67.7)	1134 (64.2)	
Ethnicity			0.053
White	23503 (87.9)	1516 (85.8)	
Black	2590 (9.7)	197 (11.1)	
Other	408 (1.5)	36 (2.0)	
Unknown	232 (0.9)	18 (1.0)	
Age			0.548
<50	4712 (17.6)	310 (17.5)	
50–59	8937 (33.4)	580 (32.8)	
60–69	6830 (25.5)	478 (27.1)	
70+	6254 (23.4)	399 (22.6)	
Charlson comorbidity score			0.605
0	21416 (80.1)	1429 (80.9)	
1	3427 (12.8)	(12.0)	
2+	1890 (7.1)	(7.1)	
Demographic area			0.064
Metropolitan	22720 (85.0)	1536 (86.9)	
Urban	3580 (13.4)	210 (11.9)	
Rural	433 (1.6)	21 (1.2)	
Income			0.010
<$38,000	5114 (19.1)	386 (21.8)	
$38,000–$47,999	6554 (24.5)	446 (25.2)	
$48,000–$62,999	7091 (26.5)	458 (25.9)	
$63,000 +	7974 (29.8)	477 (27.0)	
Percent without high school diploma			<0.01
21%+	4516 (16.9)	343 (19.4)	
13.0–20.9%	7275 (27.2)	520 (29.4)	
7.0–12.9%	8641 (32.3)	536 (30.3)	
<7.0%	6301 (23.6)	368 (20.8)	
Insurance status			<0.01
Not insured	1472 (5.5)	129 (7.3)	
Private insurance/managed care	12007 (44.9)	702 (39.7)	
Medicaid	2415 (9.0)	230 (13.0)	
Medicare	10413 (39.0)	681 (38.5)	
Other Government	426 (1.6)	25 (1.4)	

**Table 2:** Predictors of clinical AJCC stage IV disease at diagnosis. Statistically significant factors from Table 1 were included in a multivariable binary logistic regression model

	Odds Ratio	95% CI	*P‐*value
Facility type			
Community Cancer Program	1.000	–	–
Comprehensive Community Cancer Program	0.916	0.779–1.078	0.292
Academic/Research Program	1.059	0.898–1.249	0.495
Integrated Network Cancer Program	0.985	0.799–1.213	0.885
Facility location			
New England	1.000	–	–
Middle Atlantic	1.391	1.072–1.805	0.013
South Atlantic	1.217	0.941–1.573	0.134
East North Central	1.231	0.949–1.598	0.117
East South Central	1.516	1.132–2.030	0.005
West North Central	1.100	0.814–1.487	0.535
West South Central	1.688	1.263–2.254	<0.01
Mountain	1.458	1.062–2.003	0.020
Pacific	1.346	1.034–1.753	0.027
Gender			
Male	1.000	**–**	**–**
Female	0.895	0.809–0.991	0.034
Insurance status			
Not insured	1.000	–	–
Private insurance/managed care	0.722	0.592–0.882	<0.01
Medicaid	1.106	0.880–1.389	0.388
Medicare	0.795	0.652–0.970	0.024
Other Government	0.675	0.434–1.052	0.082
Median income			
<$38,000	1.000	–	–
$38,000–$47,999	0.974	0.836–1.135	0.736
$48,000–$62,999	0.964	0.818–1.137	0.664
>$63,000	0.949	0.783–1.149	0.590
Percent without high school diploma			
≥21%	1.000	–	–
13.0–20.9%	1.018	0.874–1.185	0.821
7.0–12.9%	0.945	0.797–1.122	0.521
<7.0%	0.919	0.750–1.128	0.420

Odds ratios indicate the relative odds of presenting with metastatic disease compared to the reference group.

**Figure 3:**
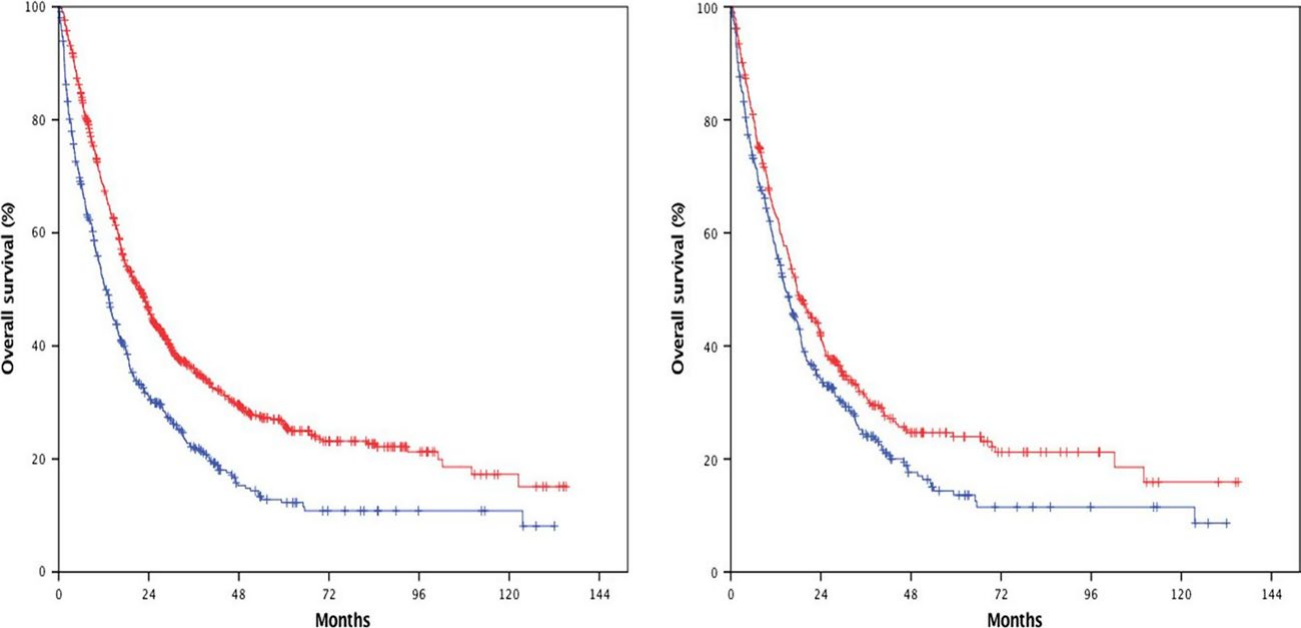
Kaplan–Meier survival curves for patients presenting with metastatic disease, stratified by receipt of pelvic radiotherapy. The median OS was 21.6 months versus 12.5 months in favor of pelvic RT in the unadjusted cohort (left, *P* < 0.01). The median OS was 17.6 months versus 14.5 months in favor of pelvic RT in the propensity‐score matched cohort (right, *P* < 0.01).

Following application of secondary exclusion criteria, the metastatic cohort consisted of 1264 patients (Fig. 1). The median OS for the metastatic cohort was 17.3 months. When stratified by receipt of pelvic radiotherapy, the median OS was 21.6 months in patients who received radiotherapy versus 12.5 months in those who did not (*P *< 0.01, Fig. 3). Factors associated with receipt of pelvic radiotherapy included treatment gender, Charlson Comorbidity Score, clinical AJCC T3 or greater disease, and receipt of chemotherapy (Table 3). On univariate analysis, factors associated with improved survival included female gender, private insurance, higher income, higher education, receipt of pelvic radiotherapy, and receipt of chemotherapy (Table 4). Contrarily, older age, increasing Charlson comorbidity score, clinical T3 or greater disease, and Tx disease were associated with worsened survival. A multivariable Cox proportional hazards model confirmed female sex, possession of a private insurance plan, receipt of pelvic radiotherapy, and receipt of chemotherapy as independent predictors of improved survival; furthermore, when accounting for other variables possession of a Medicare insurance plan was associated with improved survival, which was not observed when insurance status was analyzed in the univariate setting (Table 4). Additionally, multivariable analysis confirmed that older age and higher Charlson comorbidity score were associated with worsened survival.

**Table 3:** Baseline patient characteristics for the unadjusted and propensity‐score matched metastatic cohorts, stratified by receipt of pelvic radiotherapy

	Unadjusted Cohort (n = 1264)			PSM Cohort (n = 730)		
	No Pelvic RT N (%)	Pelvic RT N (%)	*P‐*value	No Pelvic RT N (%)	Pelvic RT N (%)	*P‐*value
Facility type			0.073			0.630
Community Cancer	51 (11.0)	94 (11.7)		45 (12.3)	42 (11.5)	
Comprehensive Community	181 (39.2)	359 (44.8)		142 (38.9)	153 (41.9)	
Academic/Research	184 (39.8)	261 (32.5)		146 (40.0)	132 (36.2)	
Integrated Network Cancer	46 (10.0)	88 (11.0)		32 (8.8)	38 (10.4)	
Gender			0.024			0.702
Male	183 (39.6)	267 (33.3)		134 (36.7)	139 (38.1)	
Female	279 (60.4)	535 (66.7)		231 (63.3)	226 (61.9)	
Insurance status			0.328			0.431
Not insured	33 (7.1)	75 (9.4)		29 (7.9)	28 (7.7)	
Private insurance	182 (39.4)	319 (39.8)		158 (43.3)	150 (41.1)	
Medicaid	64 (13.9)	97 (12.1)		44 (12.1)	40 (11.0)	
Medicare	179 (38.7)	296 (36.9)		130 (35.6)	136 (37.3)	
Other Government	4 (0.9)	15 (1.9)		4 (1.1)	11 (3.0)	
Income			0.683			0.615
<$38,000	91 (19.7)	180 (22.4)		66 (18.1)	78 (21.4)	
$38,000–$47,999	118 (25.5)	198 (24.7)		89 (24.4)	89 (24.4)	
$48,000–$62,999	127 (27.5)	220 (27.4)		113 (31.0)	100 (27.4)	
$63,000+	126 (27.4)	204 (25.4)		97 (26.6)	98 (26.8)	
Percent without high school diploma			0.477			0.349
≥21%	81 (17.5)	165 (20.6)		61 (16.7)	77 (21.1)	
13.0–20.9%	143 (31.0)	232 (28.9)		109 (29.9)	111 (30.4)	
7.0–12.9%	137 (29.7)	246 (30.7)		111 (30.4)	94 (25.8)	
<7.0%	101 (21.9)	159 (19.8)		84 (23.0)	83 (22.7)	
Charlson comorbidity Score			0.012			0.898
0	355 (76.8)	670 (83.5)		291 (79.7)	292 (80.0)	
1	66 (14.3)	86 (10.7)		46 (12.6)	48 (13.2)	
2+	41 (8.9)	46 (5.7)		28 (7.7)	25 (6.8)	
Ethnicity			0.780			0.835
White	398 (86.1)	689 (85.9)		318 (87.1)	312 (85.5)	
Black	46 (10.0)	89 (11.1)		33 (9.0)	38 (10.4)	
Other	11 (2.4)	15 (1.9)		10 (2.7)	9 (2.5)	
Unknown	7 (1.5)	9 (1.1)		4 (1.1)	6 (1.6)	
Age			0.839			0.588
<50	87 (18.8)	140 (17.5)		66 (18.1)	69 (18.9)	
50–59	148 (32.0)	265 (33.0)		126 (34.5)	117 (32.1)	
60–69	119 (25.8)	219 (27.3)		97 (26.6)	89 (24.4)	
70+	108 (23.4)	178 (22.2)		76 (20.8)	90 (24.7)	
Demographic area			0.865			0.657
Metropolitan	402 (87.0)	692 (86.3)		315 (86.3)	313 (85.8)	
Urban	54 (11.7)	101 (12.6)		46 (12.6)	50 (13.7)	
Rural	6 (1.3)	9 (1.1)		4 (1.1)	2 (0.5)	
AJCC T Stage			<0.01			0.542
≤T2	95 (20.6)	210 (26.2)		86 (23.6)	86 (23.6)	
T3–T4	166 (35.9)	428 (53.4)		148 (40.5)	161 (44.1)	
Tx	201 (43.5)	164 (20.4)		131 (35.9)	118 (32.3)	
Abdominoperineal resection			0.025			0.700
No	438 (94.8)	780 (97.3)		350 (95.9)	352 (96.4)	
Yes	24 (5.2)	22 (2.7)		15 (4.1)	13 (3.6)	
Chemotherapy			<0.01			0.350
No	168 (36.4)	67 (8.4)		76 (20.8)	66 (18.1)	
Yes	294 (63.6)	735 (91.6)		289 (79.2)	299 (81.9)	

**Table 4:** Cox proportional hazards analysis. Statistically significant predictors of overall survival on univariate analysis were incorporated into a multivariable model

	Univariate			Multivariable		
	HR	95% CI	*P‐*value	HR	95% CI	*P‐*value
Facility location						
New England		Reference				
Middle Atlantic	0.857	0.603–1.218	0.389	–	–	–
South Atlantic	1.068	0.763–1.495	0.700	–	–	–
East North Central	1.086	0.769–1.534	0.639	–	–	–
East South Central	0.949	0.646–1.395	0.791	–	–	–
West North Central	0.842	0.565–1.255	0.398	–	–	–
West South Central	0.960	0.653–1.411	0.834	–	–	–
Mountain	0.927	0.607–1.415	0.724	–	–	–
Pacific	1.063	0.748–1.509	0.734	–	–	–
Facility type						
Community Cancer Program		Reference				
Comprehensive Community	0.953	0.769–1.181	0.661	–	–	–
Academic/Research	0.835	0.668–1.042	0.110	–	–	–
Integrated Network	0.978	0.743–1.287	0.875	–	–	–
Gender						
Male		Reference				
Female	0.698	0.610–0.798	<0.01	0.795	0.691–0.915	<0.01
Insurance status						
Not insured		Reference				
Private insurance	0.625	0.491–0.796	<0.01	0.655	0.512–0.837	<0.01
Medicaid	1.026	0.777–1.353	0.858	0.955	0.720–1.267	0.751
Medicare	0.959	0.755–1.218	0.732	0.695	0.529–0.912	<0.01
Other Government	1.160	0.658–2.045	0.607	0.932	0.523–1.662	0.812
Income						
<$38,000		Reference				
$38,000–$47,999	0.972	0.805–1.174	0.771	0.948	0.773–1.163	0.608
$48,000–$62,999	0.863	0.716–1.041	0.125	0.895	0.720–1.113	0.319
$63,000+	0.815	0.673–0.986	0.035	0.868	0.667–1.129	0.292
Percent without high school diploma						
≥21%		Reference				
13.0–20.9%	1.058	0.876–1.278	0.557	1.096	0.893–1.345	0.380
7.0–12.9%	0.989	0.819–1.194	0.906	1.091	0.871–1.366	0.449
<7.0%	0.803	0.651–0.990	0.040	0.981	0.742–1.297	0.894
Charlson comorbidity score						
0		Reference				
1	1.303	1.071–1.585	0.008	0.995	0.810–1.222	0.961
2+	1.514	1.184–1.936	0.001	1.439	1.117–1.854	<0.01
Ethnicity						
White		Reference				
Black	1.018	0.821–1.263	0.868	–	–	–
Other	1.443	0.915–2.276	0.115	–	–	–
Unknown	0.908	0.487–1.695	0.763	–	–	–
Age						
<50		Reference				
50–59	0.961	0.791–1.168	0.692	1.062	0.871–1.295	.550
60–69	1.051	0.859–1.287	0.627	1.149	0.930–1.421	.199
70+	1.591	1.299–1.947	<0.01	1.597	1.236–2.062	<0.01
Demographics						
Metropolitan		Reference				
Urban	1.335	1.103–1.616	<0.01	1.336	1.093–1.632	<0.01
Rural	1.558	0.901–2.697	0.113	1.457	.831–2.554	.189
AJCC T Stage						
≤T2		Reference				
T3–T4	1.245	1.054–1.470	0.010	1.314	1.109–1.557	<0.01
Tx	1.403	1.170–1.682	<0.01	1.311	1.088–1.579	<0.01
Pelvic radiotherapy						
No		Reference				
Yes	0.630	0.551–0.721	<0.01	0.770	0.661–0.897	<0.01
Abdominoperineal resection						
No		Reference				
Yes	0.941	0.665–1.332	0.732	–	–	–
Chemotherapy						
No		Reference				
Yes	0.351	0.300–0.410	<0.01	0.457	0.381–0.549	<0.01

Propensity‐score matching was then applied to generate a 1:1 paired cohort of 730 patients to further control for known confounders. In contrast to the unadjusted cohort, patient and treatment characteristics were well balanced without statistically significant proportional differences when stratified by receipt of radiotherapy (Table 3). Comparable to the initial cohort, the median overall survival was 15.8 months in this cohort. When stratified by receipt of pelvic radiotherapy, the median OS was 17.6 months in patients who received radiotherapy versus 14.5 months in those who did not (*P *< 0.01).

## Discussion

In this study of national, prospectively collected data from patients with anal cancer, the importance of social determinants in the diagnosis of metastatic anal cancer is evident. While income, education, and ethnicity were not independent risk factors for a stage IV diagnosis, patients without insurance were more likely to present with metastatic disease than those with a private insurance plan or a Medicare insurance plan. While there is a relative higher incidence of anal cancer in the black population than found in other ethnicities 1, this disparity was not mirrored by a higher rate of metastatic disease at presentation. Early symptoms, which can include bleeding, incontinence, and anorectal pain are at least in part responsible for the generally low rates of metastatic disease at presentation. However, previous data suggests that uninsured patients are less likely to seek medical attention than their insured counterparts, even with bothersome or potentially life‐threatening symptoms 17. It is possible that lack of insurance resulted in a delayed diagnosis leading to higher rates of metastatic disease. Similarly, there was a lower rate of metastatic disease in the female population observed in this study and prior research has demonstrated that women are more likely to seek medical attention than their male counterparts 18. Finally, these results indicate that the rates of metastatic disease also varied by geographic location. Although previous studies have demonstrated that the incidence and death rates of common cancers are known to differ by geography 19, the underlying cause of the observed geographic variance observed in this study is not immediately apparent. However, the presence of Texas in this region, which is the second largest state by population 20 and has the largest proportion of uninsured patients in the United States 21, may be the underlying driver of these findings.

The management of metastatic disease in patients with anal cancer remains challenging. Given the location of the primary tumor, local treatment may be necessary to palliate or prevent morbid progression; however, the overall effect of such treatment is unknown. Recently, presented data suggests that chemoradiotherapy for squamous cell cancers of the esophagus in patients with metastatic disease is associated with an overall survival benefit compared to chemotherapy alone 14. A similar effect was observed in this patient cohort, a finding that was confirmed by multivariable analysis in the unadjusted setting, as well as a propensity‐score matched subset with well balanced confounders. Given the relative radiosensitivity of anal squamous cell cancers 2 and propensity for devastating morbidity, consideration of local treatment should be considered in patients with metastatic anal cancer, particularly in those for whom chemotherapy will be administered.

This study has several limitations. Although majority of cancer cases are captured by the NCDB, it is a hospital‐based registry rather than a population‐based registry like the smaller Surveillance, Epidemiology, and End Results (SEER) database. Data is captured from hospitals accredited by the Committee on Cancer, a program of the American College of Surgeons. Consequently, although roughly 70% of all cancer diagnoses are evaluable within the NCDB, the sample may not truly represent the national patient population. While data is collected prospectively, this analysis is retrospective, with all of the limitations inherent in such an analysis. Furthermore, although the patient and treatment information that is collected is quite comprehensive, certain critical components are missing from this analysis. Viral infections, such as the human papilloma virus (HPV) and human immunodeficiency virus (HIV), are intimately associated with anal cancer and convey a drastically increased risk of eventual diagnosis 22, 23. No information regarding these infections is presented in the NCDB, although a patient with clinical acquired immune deficiency syndrome (AIDS) would fall within the Charlson Comorbidity Score 2+ classification, a group that represented only 7.1% of patients in this analysis. Finally, outcome data is limited—excluding surgical mortality, the only evaluable outcome is overall survival. While a profound benefit was noted for metastatic patients receiving pelvic radiotherapy, it is possible that the impact on local control may be far greater. Additionally, as no information regarding toxicity is currently available within the NCDB, the potential adverse symptomatic effect of palliative radiotherapy cannot be determined. Given the morbidity of untreated local progression, it is reasonable to surmise that local therapy may improve patient quality of life. However, without clinical data in support of this supposition, consideration of palliative radiotherapy should be undertaken on a case‐by‐case basis.

In summary, this analysis demonstrates that lack of healthcare insurance coverage is associated with a higher incidence of metastatic and incurable anal cancer. Additionally, there was improved survival in metastatic anal cancer patients who received radiation therapy, suggesting a role for local treatment even in the presence of systemic disease. Further prospective research is warranted to validate this finding.

### Disclaimer

The NCDB is a joint project of the Commission on Cancer of the American College of Surgeons and the American Cancer Society. The data used in the study are derived from a deidentified NCDB file. The American College of Surgeons and the Commission on Cancer have not verified and are not responsible for the analytic or statistical methodology employed, or the conclusions drawn from these data by the investigator.

## Conflicts of Interest

None declared.
